# SAPPHIRE: a neural network based classifier for σ70 promoter prediction in Pseudomonas

**DOI:** 10.1186/s12859-020-03730-z

**Published:** 2020-09-22

**Authors:** Lucas Coppens, Rob Lavigne

**Affiliations:** Laboratory of Gene Technology, Department of Biosystems, KU Leuven, Kasteelpark Arenberg 21, Box 2462, 3001 Leuven, Belgium

## Abstract

**Background:**

In silico promoter prediction represents an important challenge in bioinformatics as it provides a first-line approach to identifying regulatory elements to support wet-lab experiments. Historically, available promoter prediction software have focused on sigma factor-associated promoters in the model organism *E. coli.* As a consequence, traditional promoter predictors yield suboptimal predictions when applied to other prokaryotic genera, such as *Pseudomonas,* a Gram-negative bacterium of crucial medical and biotechnological importance.

**Results:**

We developed *SAPPHIRE,* a promoter predictor for σ70 promoters in *Pseudomonas.* This promoter prediction relies on an artificial neural network that evaluates sequences on their similarity to the − 35 and − 10 boxes of σ70 promoters found experimentally in *P. aeruginosa* and *P. putida*. *SAPPHIRE* currently outperforms established predictive software when classifying *Pseudomonas* σ70 promoters and was built to allow further expansion in the future.

**Conclusions:**

*SAPPHIRE* is the first predictive tool for bacterial σ70 promoters in *Pseudomonas*. SAPPHIRE is free, publicly available and can be accessed online at www.biosapphire.com. Alternatively, users can download the tool as a Python 3 script for local application from this site.

## Background

Promoter prediction in prokaryotes has received a lot of attention over the past two decades, to enhance the understanding and construction of gene regulatory networks [14]. Several tools implementing diverse algorithms, ranging from simple motif searches to complex machine learning techniques such as neural networks and support vector machines, have been developed and made available to the scientific community [7, 10, 11]. A unique approach was proposed in a paper studying thermodynamic stability of DNA as a feature for promoter prediction, rather than DNA motifs [9]*.* A key limitation to many of these bacterial promoter prediction tools is their bias towards σ factors from *Escherichia coli*. Consequently, application of these tools to other bacterial species yields suboptimal promoter predictions.

We here describe the Sequence Analyser for the Prediction of Prokaryotic Homology Inferred Regulatory Elements (*SAPPHIRE*), a tool developed to predict σ70 promoters in *Pseudomonas aeruginosa* and *Pseudomonas putida*. *P. aeruginosa* strains are opportunistic, multidrug-resistant pathogens of the highest global priority [8, 15]. *Pseudomonas putida* is a promising bacterial chassis for synthetic biology applications with an industrial scope [5, 6]. Currently, 4660 and 127 sequenced genomes of *P. aeruginosa* and *P. putida* are available on the Pseudomonas Genome Database, respectively [16]. Yet only a small fraction of these genomes contains annotated promoters, illustrating the shortage of available tools for promoter annotation.

The underlying model of SAPPHIRE combines the strong predictive power of a fully-connected artificial neural network with the traditional approach of relying on − 35 and − 10 boxes of σ70 promoter sequences, which are the distinguishing features of σ70 promoters and have been thoroughly analysed using information theory [12].

## Implementation

### Data

SAPPHIRE was trained using a dataset of 170 unique Pseudomonas σ70 promoters (Additional file 1). Ninety four of these sequences were taken from experimentally validated *P. aeruginosa* and *P. putida* σ70 promoters [3]. The 76 remaining σ70 promoters were retrieved from the NCBI Nucleotide database (database query for annotated *P. aeruginosa* and *P. putida* sequences containing keywords “minus_35_signal” and “minus_10_signal”).

Sixteen thousand background sequences were randomly extracted from intergenic genomic regions that were not annotated as promoters in the *P. aeruginosa* PAO1 genome. Extraction of background sequences from these Pseudomonas sources provided a biologically meaningful set of negative examples for training as these intergenic sequences compete with σ70 promoters for RNAP binding in the cytoplasm. The strong imbalance between positive and negative examples in the dataset is justified by a similar ratio of promoter to non-promoter sequences in bacterial genomes. Prior to training, the complete dataset of positive and negative examples was randomly divided in a training set and test set using a 9:1 ratio.

To ensure the quality of the data, the negative sequences were positively verified to contain no obvious sequence motifs that could unwillingly be learned by the neural network, using the MEME suite [1]. Furthermore, the degree of conservation of the 12 nucleotides in the − 10 and − 35 boxes of between any two of the positive and any two of the negative sequences were found to be 5.6/12 and 3.2/12 on average. For the positive sequences, this degree of conservation, averaging below 50%, confirms satisfactory independence of the training sequences. This is also the case for the negative examples, with a degree of conservation that is not significantly higher than what would be expected if they were randomly generated sequences (3/12, 25%). The positive and negative examples contained average GC-contents of 47 and 61%, which is a notable difference and a feature that could possibly be unwillingly incorporated in the neural network. However, this low GC-content was not compensated for, as it provides a potentially valuable distinguishing characteristic of promoter regions for a neural network, as illustrated by the consensus sequence TTGACA-TATAAT (17% GC).

### Features

From the sequences that were collected to train the neural network, only the six nucleotides in both the − 35 and − 10 boxes were used as features for training. For each sequence, these twelve nucleotides were one-hot encoded, providing data features that can be passed to a neural network.

### Neural network architecture

**Fig. 1:**
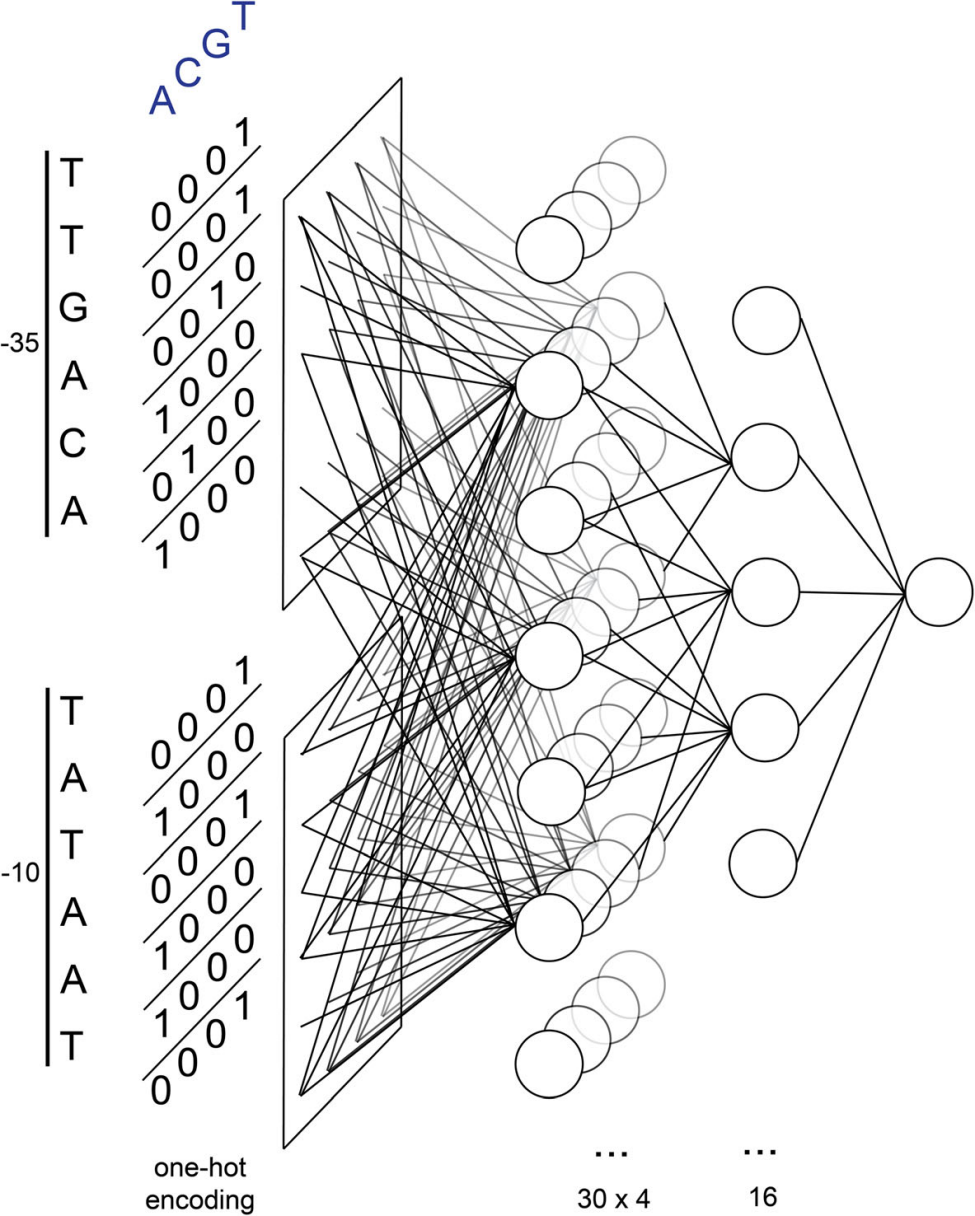
Schematic overview of the SAPPHIRE artificial neural network

The core architecture of the neural network (Fig. 1) consists of two consecutive fully connected layers, feeding into a single-node third layer. The first layer contains 30 × 4 nodes, the depth of 4 being a remnant of the one-hot encoding of the input sequences. The second layer (16 nodes) flattens the two dimensions into a single dimension. A single node in the third layer presents the output of the network. The activation function of the nodes in the first/second layer is the rectified linear unit (ReLU). A sigmoid activation function was chosen for the final node, as it is well-suited for binary classifiers due to its output value between 0 and 1. The choices of all neural network design parameters are the result of optimization by manual tuning of the network, using the performance of fivefold cross-validation on the training set as a measure.

### Significance estimation

We introduced a framework to estimate the *p*-value of positive hits, providing a measure for the significance of sequences classified as promoters. We defined the p-value of a hit as the probability of a randomly generated sequence to produce a value at the output node of the neural network equal to or greater than the output value produced by that positive hit. In order to estimate this probability, an estimation of the posterior probability distribution of the output value of the neural network was generated using Monte Carlo sampling (10 million random sequences). An approximation of the *p*-value is then correspondingly calculated as the fraction of random output values in this distribution equal to or greater than the output value produced by a new positive hit.

## Results

### Benchmarking

Due to the imbalanced nature of the dataset, accuracy was expected to accommodate a bias towards specificity and mask the minority-class performance (true positive rate). Therefore, accuracy would not provide a suitable metric in this case. To avoid this bias, sensitivity (true positive rate) and specificity (true negative rate) were chosen as metrics for evaluation. After optimization of the design parameters of the network, the model yielded values of 76.6 and 88.1% for mean sensitivity and mean specificity respectively in fivefold cross-validation on the training set. These results indicate a good over-all performance of the model. Additionally, the model for *Pseudomonas* σ70 promoters specifically was compared to two established online tools for bacterial promoter prediction, BPROM [13] and CNNPromoter_b [14]. Both the complete and test dataset were analyzed using SAPPHIRE, BPROM and CNNPromoter_b (Table 1).

**Table 1:** Benchmarking of SAPPHIRE against online available promoter prediction tools

	Test set		Complete dataset	
Tool	Sensitivity	Specificity	Sensitivity	Specificity
BPROM	23.5%	78.8%	18.8%	79.9%
CNNPromoter_b	23.5%	86.9%	18.8%	87.2%
SAPPHIRE	88.2%	82.9%	87.1%	82.0%

Sensitivity and specificity for each tool and both the test set and complete dataset.

The results show that SAPPHIRE outperforms both BPROM and CNNPromoter_b in terms of sensitivity and shows similar performance in terms of specificity, scoring slightly better than BPROM and slightly worse than CNNPromoter_b. The large difference in sensitivity implies that SAPPHIRE effectively better distinguishes σ70 promoters from background sequences in Pseudomonas. Furthermore, the notably poor score of 18.8% for sensitivity on the complete dataset (32 out of 170 promoter sequences detected) from both BPROM and CNNPromoter_b corroborates the need for *Pseudomonas* specific promoter prediction tools.

### Case study: uncovering σ70 promoters in *Pseudomonas* phages

**Fig. 2:**
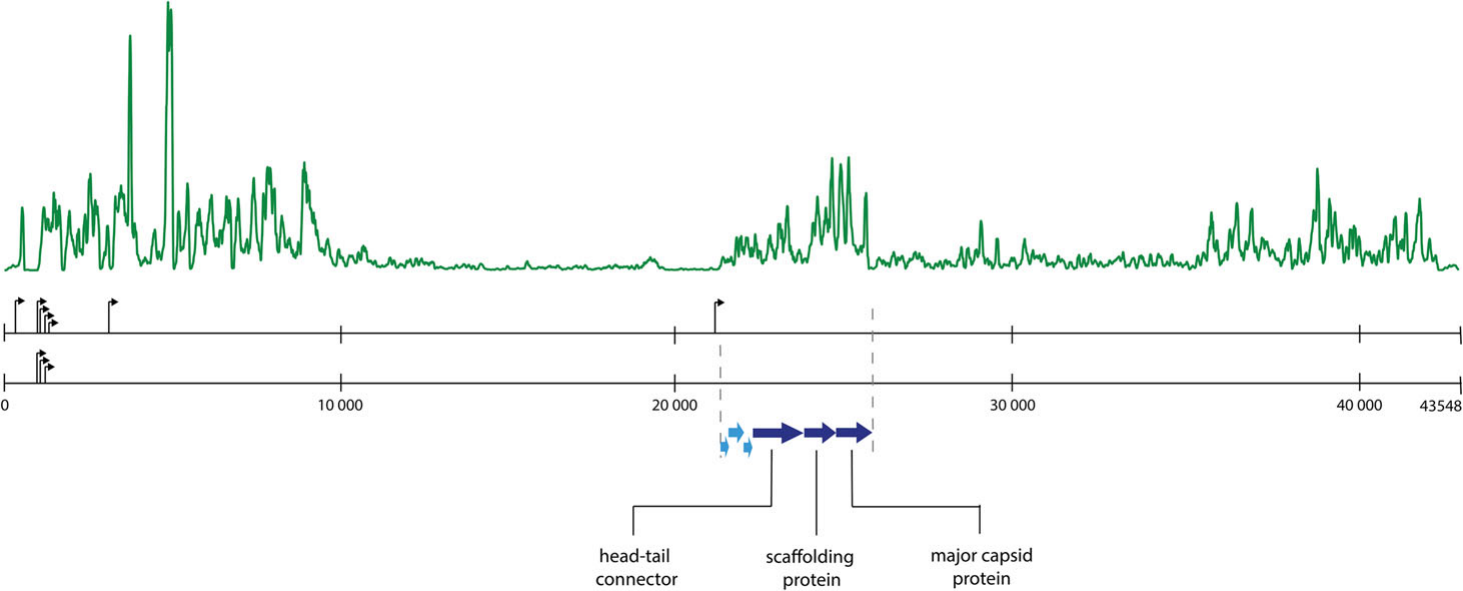
Top: Transcriptomic landscape of LUZ19 during early infection. Middle: Arrows indicate the σ 70 promoters discovered by SAPPHIRE, compared to previously annotated σ 70 promoters. Bottom: location of the three structural protein coding genes are indicated

To further verify the quality of SAPPHIRE as a predictive tool, we applied it to scan the intergenic regions on the genome of *Pseudomonas aeruginosa* bacteriophage LUZ19. The family of the *Autographivirinae,* of which LUZ19 is a member, is known to rely on the host’s σ70 transcriptional apparatus during early infection [4]. Correspondingly, three σ70 promoters at genomic locations 913, 982 and 1147 have already been identified and annotated early on the LUZ19 genome. Using SAPPHIRE with a *p*-value cutoff of 2*10^− 4^, four additional promoters could be identified in LUZ19’s intergenic regions. Figure 2. shows these promoters at their specific locations on the LUZ19 genome, along with the transcriptomic landscape of LUZ19 during early infection, as determined by RNA-seq [2]. Each of the predicted sequences correlates to the start of a transcribed genomic region, substantiating the ability of SAPPHIRE as a tool to identify new σ70 promoters. Remarkably, one of the identified promoters on LUZ19 seems to drive the early expression of a cluster of genes including the head-tail connector, scaffolding protein and major capsid protein, structural phage genes which are generally thought to be expressed under a phage RNAP-specific promoter during late infection in members of the *Autographivirinae.*

**Fig. 3:**
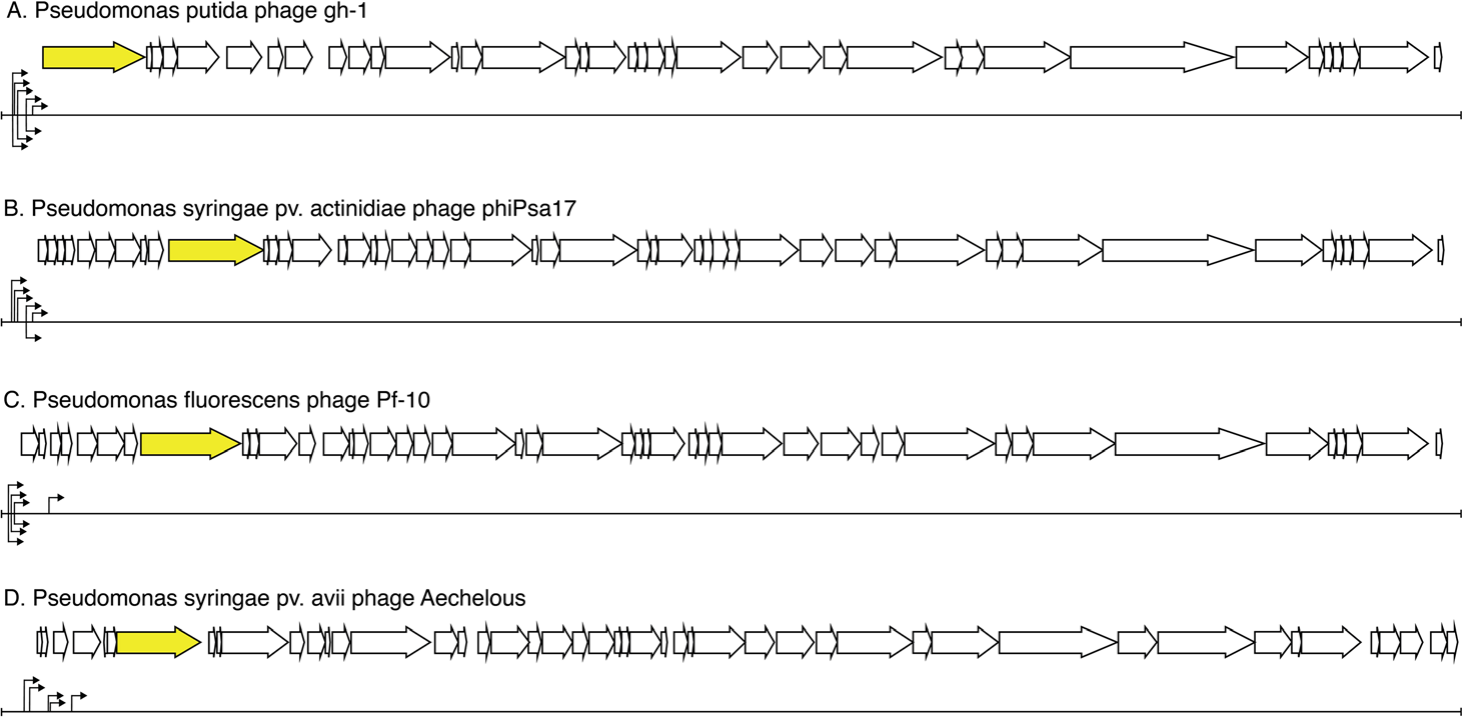
Coding sequences and sig70 promoters on phages gh-1 phiPsa17. Upwards arrows indicate σ 70 promoters predicted by SAPPHIRE. Downward arrows indicate annotated σ70 promoters. Phage RNA polymerases are highlighted in yellow

Furthermore, the intergenic regions of the genomes of four representative members of the *Autographivirinae,* infecting a variety of *Pseudomonas* species were subjected to the *SAPPHIRE* software (Fig. 3). In addition to identifying the promoters that had previously been annotated on these genomes, *SAPPHIRE* predicts additional promoters on these genomes. These newly predicted promoter sequences deviate from the − 35 and − 10 consensus sequences, offering an explanation for why they had not yet been annotated. However, all of the newly discovered promoter sequences are consistent with the genome organization architecture of this clade of viruses. Indeed, *Autographivirinae,* are known to encode σ70 promoters at the left end of their genomes, driving expression of a phage-encoded RNA polymerase that subsequently transcribes the remainder of phage genes. These biologically consistent findings on phage genomes suggest that *SAPPHIRE* provides suitable predictions for multiple members across the *Pseudomonas* genus.

## Conclusion

*SAPPHIRE* is the first online predictive program that specifically targets σ70 promoters in *Pseudomonas* and its viruses. This new tool combines the traditional approach to promoter prediction of searching − 35 and − 10 boxes with the strong predictive capabilities offered by a neural network architecture. Our stringently selected dataset and a focused number of nucleotide features used by the neural network ensured a model with high sensitivity and specificity. In line with its initial objective, *SAPPHIRE* was shown to outperform other σ70 promoter prediction tools for *Pseudomonas*. In future, this tool can be expanded towards other sigma factors and species, depending on the availability of experimental datasets.

### Availability and requirements

**Project name:** SAPPHIRE**Project home page:** www.biosapphire.com

**Operating system(s):** Platform independent**Programming language:** Python 3.

**Other requirements:** Python3 and python packages: numpy, keras, biopython.

**License:** Creative Commons Attribution 4.0 International License (http://creativecommons.org/licenses/by/4.0/).

**Any restrictions to use by non-academics:** None.

## Supplementary information

**Additional file 1.**

## Data Availability

The dataset used during the study is available in Additional file 1.

## Abbreviations

RNAP: Ribonucleic acid polymerase
RNA-seq: RNA-sequencing
SAPPHIRE: Sequence Analyser for the Prediction of Prokaryotic Homology Inferred Regulatory Elements
ReLU: Rectified Linear Unit
