# Supplementary material for: SAPPHIRE: a neural network based classifier for σ70 promoter prediction in Pseudomonas
Source: BMC Bioinformatics. 2020 Sep 22;21:415. doi: 10.1186/s12859-020-03730-z (PMC7510298; doi:10.1186/s12859-020-03730-z)
Supplement: Supplementary file 1 — Additional file 1. [file 12859_2020_3730_MOESM1_ESM.docx]

**SUPPLEMENTARY DATA**

***SAPPHIRE*: A neural network based classifier for σ70 promoter prediction in Pseudomonas**

**Promoter sequences used to train *SAPPHIRE***

**1 *P. aeruginosa* promoter sequences from Domínguez-Cuevas and Marqués (2004)**

>amie

-----GATTACATAACGTTACACGAACCTTGACAGCCCCTTCCGACGGGGCTTATAAGTGGCGCCA---

>uvrA

-----CCTCGCGGCACAGGCGCGCCACTTTGCCGCGCGCTAGCGGCCAGCCGTATAATCAGCGGTT---

>hcnA2

----AAGATACCCACCTGTCATGGATCAATGTCC-GCGCCCGGCCCGACTCCTAGTGTCGCCGGCT---

>pchD2

----AATCGAAAGGCGCGGGCTTGCGCGGTCCCTGCCGCCCGCCAATGATAATAAATCTCATTTC----

>pchR

---ATGCGATCTCCGTGGATGCGGTCGATTGCCATTGTTGGGAAATGAGATTTATTATCATTGG-----

>mexR

------TCAACTATTTTGCTTATTTTAGTTGACGTTATCAACCTTGTTTCAGGTTCTGAATATGGGC--

>mexA

-------GTAAACCTAATGTAAATGTGGTTGATC-CAGTCAACTATTTTGCTTATTTTAGTTGACCTTA

>gdhB

----GAACGAATATGTCGCAGCACGGTAAGTGTT-TTGCCTTGCCGGCTCGTTAAGCTCTGTCGGA---

>lasB

-TCAAGGCTACCTGCCAGTTCTGGCAGGTTTGGC--CGCGGGTTCTTTTTGGTACACGAAAGCA-----

>arcDA

-GTAGGAAGCTATTGACGTGGATCAGCATTCAAC--AATAGCCGCTGCCGCCTAATGTCTTCAC-----

>groESL2

---GAAAAAAAAATTTCACTGCATGCCCTTGAAA--TGGCTTGTGGCGACCTTATGTAACGGTCAC---

>aguBA

----ATCCATAACACCCTCTTAAACCGCTTCAGA-GTCCGATTTTTATCGGATATAAATCCGCCCA---

>argF

---GTAACCCGCGCCTTCCAAGCCCTCCTTGTGT--TTCCGCGACATTTCCTTATAAGATCGCGCC---

>OprF

------GAAACATAGTTGGGTAAATATTGTCTCTCTATGCGGGAAGTTCTGATAAACTTGCCACCCA--

>algC

-----GTCCTGACCCTCGGTCAGTATCTTCAGGAACTCGGCGGGCAACGCACTGCCAAACCCCCTG---

>rpoD

-----TTCCCAGAGCTGAACTGGCGCCTCAGCGCAGGCATCAGGTATAATCCTCTGCTTATTTTTT---

>exoS

---GTGAAAAAACGGCGGCCAATCCTGATAGGGG-ATGGGGTTTCCCGTTCCTAGACTGGCGGAG----

>rpoH70

--CGCTGTAAAGGAACTTATACACCCGCTTGCAG--TCAGATATCCGAGTGCTACACTGCGCGAG----

>algu3

---TCATCAGAGCGGGGCGATGTAGTGCTGGAAC-TTTCTTAGACGCATCGGTTCCAAAGCAGGA----

>pigA

--ATGGCAGTCGGTCGGCATCCTTTCCTGTGACATTGAGATTCAATAACGAATATAATTGAGA------

>pigB

--GTGACTGTGGTGTGACGGTACCTGCGGTGACATGTTTTCACCGATTGCCGTATAAGCGGGA------

>ciaoAB

---AATTTGTCTCGCGACAACCGACCACAGCAGG-ACGTCTAGGCTAACGGGTAAGATTTTCGTA----

>hscA

-----CGACAAGCTGGCGCAGGAAGTGCGACAGCTGGAAGAGCGACTCGACGATTAATCCGCGCGG---

>gbuA

--CTGAACTGATTATTTGTAGCCAGCGATTTGAC-TCGCTACCGGCTGTGAACAGAATCCATCC-----

>migA

----CGATCGCCGGAACGATCGCAGGACTTCATG-ACCGACGGCCCCCTGGAACGGCTACCGGGCA---

>oprX

--ATGTCTGCTTTCTCAGCGCGGGATGTTTCGGA-GTATCCCGGAATACGGGTATGATCGGGCT-----

>var

-----AGTCCTGATAGCTGCGTCGCAAAATCAGA-TCGACCTTCATGGTCCGTCTGTTAAGCTGGAC--

>carb

---AAGGGGACCGGGCTCGGCCTCGCACTGGTGT-ATTCGATCGTGGAAGAGCATTATGGACAGA----

>hemL

----CAACTGGAACTGGCCGAGCAGGCGGTGGGC--GAGGGCGCCAGCTACGTCGCCTTCGGCCGCT--

>regA1

-----CCTGCCCCCTGGGTTTTCCGACGAAAGAC--CTTGATTCGTGGGAGGTAGGGTCGTCTCCGCT-

>carA

---GCGCTGCCGCACGTCGTCTTATTGGTGGACC-GGAATGTCGCGATTCTGTAAACTACAGCTT----

>pvdS

----GTGAGATTGGTTATTTCTTCGTAATTGACA-ATCATTATCATTCAACATAATTTGTTGCGCC---

>trpF

----TTCCGGATTGCCCGATATCGTCGGCTGACG-TCGAATCGGGCTTTTGCTACCATCGAGAATT---

>ALGR2

---GTATGAATTTTTCTGTACGGGCGTCTTGATG-GGGTCATCCCGGGGGCATAATCTGGCTTTC----

>ALGR

----GTCGCTTGTAGCACATCGAACCCGTTGGGGAGAGGGGGTTTGCGGGTCTAGTATGGGCGCA----

>RNAR4_5S

--ATTGCAAACGTAAGTGCTTGAAGTGGTTGTAA--CTTACGCGCAAGCCGGTAGAATGGCGCGG----

>AZURIN1

----TCCGGTTCGGGTTTGACCTGAATCAGTGGAACTCGGTGCCCGATCGGGCAGTCTGCTCTTT----

**2 *P. putida* promoter sequences from Domínguez-Cuevas and Marqués (2004)**

>putA

------GAAAAAATCGGGTTGCACCTGGTTGCAC-CCGAATTGTCCTACAGATACTCTTGACGCCAGC-

>ttgR

----GGAAAAAATGCTATCCGGTGGATAAATAGC-TTGCTAAGGAATATACTTACATTCATGGTTG---

>pcaR

----ATAAGCAAAAGTGTCGGCGGTCAATTGCGA-TTATCGGCCGTTTGTTCGATAATCGCACGAA---

>alk5

----CATATCGACTACACTTAAGTGTAGTTTAAA-TATTTTACACCGTAACCTATGGTGAGAATAT---

>cat

---CAAGCGGCAAGAAGGCCCTAGCAACTTGACA-GGTGAATTCGAGGCGGATGATTTTTTTTGA----

>edda

----GGATATCGATAATTAAATAACGGATATCCG-CCTGTTTCTCAATGTCGTAGCCTTAACAACA---

>edda

----GGATACAGGAGTGCAAAAAATGGCTATCTC-TAGAAAGGCCTACCCCTTAGGCTTTATGCAA---

>nahG

----ATTTATTATATATCGAGTGGTGTATTTATC-AATATTGTTTGCTCCGTTATCGTTATTAACA---

>putP

----TGCAACTTGCAAAAGAAAAATGGTTGCACC-TTCTTTGCGTTGTTGCATAGCATTCGCCGCC---

>alkB

--TATTCAGATAACTACACCAAGGAGTAGTTGAA--GCAGGCTTAAGGTGAGTGCAGTTTTACCA----

>tfdCB

---TATCACTAGCGTCGATACAAGGTATTAGACC-ATATGGCTTGGTTTTCCTAGACTGCTCCCA----

>cyaB

-GGTACCTCCGTCCTCCAAAAAAAATCATTGACT-CAGGAGTTTTTCAGCCGGATGATCGCGA------

>tol_oprL

-----GATTTTTCCGAGCCAGGCGATGAATGACA-GATCGAAAGCGACTTTTTGTGGTCCTACCGCT--

>ttgDEF

-CATGGCATGAACGGCTGTTTCGCAAAAACCACA--TAGTGATACACTATTCTGCATTGCGGGC-----

>pros

---TTCGCTTCAAACGGTAGTTTGAATAAAGGCA--TTGATATCGCCGGTGATTTGGGACAGCCTG---

>tods

---GTTGCTCATCAAAAAAGTATTCTCAGGATGA-TACGAGGGCGTATGATCTATAATGAATCCG----

>xylR_Pr1

---CGGATAAAGGGGATCTGCGTTGAGGTGTATT-TCAGTTAATCAATTGGTTAATCTTTCAGGA----

>catBCA

----TATGGTGGGAGATTCATTTGATATTGGACGGTCATCAGGGTCTCGCGCAATCCTTGAACAA----

>ph1R

---TCACTGCCGTCGATTGATCATTTGGTTGACT-TTTGCCAGATACTGAGGCCGGCTATAGGGA----

>dnaA

---TCGTGGTTCCTAACGTGTCCACATGTGGATA-ACTGAACGCTCGACCGGTACAATGGCGGTT----

>catR

------CGTCGTTTCGGCCAAGCATCCATCGCCT-TCTTCACTGGGGTCCAATATCATCTTCCTCTCG-

>ttgGH1

----CCGCGGCTCAGGCTTGCTTGCGTCAAGAGT-ATCACATAATGCTACACTCTACCGCATTACG---

>ttgV_W

------CTATTGCTGAATCGTAATGCGGTATAGT-GTAGCATTATGTGATACTCTTGACGCAAGCAAG-

>phew

----TAAAGGTAAGCTTGAACAATAGCACCCATG-GCAACTGCTTGACGAACTCTGCCCGTACGAC---

>rpoD

-----CCCGCACAAACGCCGACCTCATCTGGCGCGTGAGGCCCATGCTCGGGTATAATCCTCGGCTT--

>pcaIJ

----GTGACGAATAACCACCAGAACTGCTCGCAC-ATCGCACAACAGTTCGATAATCGCACAAATTC--

>nahA

------AAACAAATTCAACTATGCTTTATTGACA-AATAAAAGCACGCTCACCATCATCGCGAATACA-

>phaG

-AAAGTCACATTACTTACCGAACGGCACTTGCGC-GATCCCCAACCCACTGCTTGAATCCAAA------

>nahR

--TAATAACGATAACGGAGCAAACAATATTGATA--AATACACCACTCGATATATAATAAATCAT----

>todX

----ATAAACCCATAAGCCAAAAAACAATATTTC--CAGGGCGTGATTGTAATACTGTGCGTGGTC---

>oprL

--GAAACAAGTCTTGATGAGTCTCCCACTGTTGC-GCCAAACGGCACCATGCTAATCTACGCCA-----

>bkdA

--CAATCAACTTGAGAGAAAAATTCTCCTGCCGG-ACCACTAAGATGTAGGGGACGCTGACTTA-----

>xylR_Pr2

--ATTTCAGTTAATCAATTGGTTAATCTTTCAGG-ACCACCTAAGCAAATGCTAAAGTGGCAGA-----

>benA

---TGGATAACACTCTGCACAATCCGGATAGCTC-CCCGCCAGTCGTCTCCCTAACCTGACCAGG----

>ttgaBC

-----TACCTGAGTACCACCCAGCAGTATTTACAAACAACCATGAATGTAAGTATATTCCTTAGCA---

>ttgT

-----GACAAGAGAGATCAGTCGCTCGGCTGGCCTGATCGTTCACTTGCCATTAGAGTGGTGGCCA---

>ropH

-----CGGCTTCCGATGGTCTGTCGCTCTTGATCGGAGCAGTGTTGTTGGGGTATATCGGTGCATG---

>prsA

----GGCGCTTTAAATGGCGACCGCCCGGTCACC-GAGCCTTGACAGAAGCAGGGCTGAAACGTAT---

>xylS_Ps2

----ATTTTCATCGACTTGGCGCCTTTCTACATCACACCAAGCAGCCCACATTAAAATAAGAGAA----

**3 *P. aeruginosa* promoter sequences retrieved from NCBI Nucleotide**

>PSERHTR

ATTTTGCCGTATCGGCAAGGCTGCGCGCTTGACA-GCGTCATACCCCGGGCCAATTCTGCTGTGATGCA

>EF138817

TGCTCACAGCCAAACTATCAGGTCAAGTCTGCTTTTATTATTTTTAAGCGTGCATAATAAGCCCTACAC

>PAU49151

GTCATCAGAGCGGGGCGATGTAGTGCTGGAACTT--TCTTAGACGCATCGGTTCCAAAGCAGGATGCCT

>PAU49151

AAACTCGTGACGCATGCTTGGAGGGGAGAACTTT--TGCAAGAAGCCCGAGTCTATCTTGGCAAGACGA

>X52261

CCGTTCGGAACATTCTTCCCGTCGGGGGTAGGCG-AGCGACTTGCCCTGTGGAATAATACTGTCTACTT

>AM261760

TGTCCCCAAATTTTAGCGGCTAAAGGTGTTGACGAGGGATAGAAAGTTTAGCTAAACTTCTTCCATCGA

>AM261760

ATCCCCTCAATTGTTTAGCTAAAATTGCTTGACAAGTTAGGGCATTATGCCCTATTCTTGTTTTGAGGC

>AM261760

CCGCCGCAAATTGTTTAGCTAAATTTCCTTGACTATCTAGGGCATAATGCCCTAATATAGCAATCCAAG

>AM261760

ATTGCCCGCTCCACGGTTTATAAAATTCTTGAAG-ACGAAAGGGCCTCGTGATACGCTTATTTTTATAG

>AM261760

TCAGACATAGTAAAACGGCTTCGTTTGAGTGTCCATTAAATCGTCATTTTGGCATAATAGACACATCGT

>AM261760

AACGAAGCCGTTTTACTATGTCTGATAATTTATA-ACATTTCGGACGGTTGCAAAAATGTTACTAAATG

>AM261760

GCCGGCGGCATAGTTTAGCTAAATTTGCTTGACAGGCTAGGGCATAATGCCCTAATATTGGTCTTGAGG

>AM261760

ACTGTATTCGGCTGCAACTTTGTCATGCTTGACACTTTATCACTGATAAACATAATATGTCCACCAACT

>AM261760

CGCTGGTCCGATTGAACGCGCGGATTCTTTATCACTGATAAGTTGGTGGACATATTATGTTTATCAGTG

>AM261760

GCCCGCAAAATTTTAGCCGCTAAAGTTCTTGACAGCGGAACCAATGTTTAGCTAAACTAGAGTCTCCTT

>AM261760

CGTCGTGCGGTTCGATTAGCTGTTTGTCTTGCAG-GCTAAACACTTTCGGTATATCGTTTGCCTGTGCG

>AM261760

GTCAGAATAGAATCCGCTTTCACATTCTTTGACA-CATGCTTGCCAAGGTCATAGATTTCAGCCTGACA

>AM261760

ATGACGTTTTCTAAGCCCTACCAGGCGTTTGACT-ATTAACTCCAAGGAGTAACTTATGAAGAAGCTCG

>AM261760

CAATGAGTCGTCCGCTACCTTCCTCGGCTTGTCCTTTCGAATTTGAATTGGATAGCGTAACCTTACTTC

>AM261760

TGTCATTTTCAGAAGACGACTGCACCAATTGACG-GGGCGTAACGCCAGGTGTGCAGTCGGCTCCTGAC

>AM261760

TTTCCCGACCTTAATGCGCCTCGCGCTGTAGCCT-CACGCCCACATATGTGCTAATGTGGTTACGTGTA

>AM261760

TGGCAAATAACGGTCAAACATCGTGGCGTTGACA-ACGTGCCTGGATCTGGCTACACTATGCGGCCACC

>AM261760

CAATAAAGTCTTAAACTAGACAGAATAGTTGTAA-ACTGAAATCAGTCCAGTTATGCTGTGAAAAAGCA

>AM261760

CATGCACGATTTGTAATAACAGAGTGTCTTGTAT--TTTTAAAGAAAGTCTATTTAATACAAGTGATTA

>AM261760

GCCTAGAGATGCTTGTTTACCGGTAGAGTTTTAA-TTTAATGCTAAATAAATTAAAATGTTATGAGTTC

>AM261760

GAGCGCGAGCAAAAGCGGCTAAACGGCTTCGATA-TACCGAAGCATGCACGATATACTGATTATGTTGG

>AM261760

TTCGGTATATCCATCCTTTTTCGCACGATATACA-GGATTTTGCCAAAGGGTTCGTGTAGACTTTCCTT

>AM261760

CAAAATCCTGTATATCGTGCGAAAAAGGATGGAT--ATACCGAAAAAATCGCTATAATGACCCCGAAGC

>AM261760

GCATCGTCCACGCAGATAGCGGCAGCATTTACCAACAGGTGGGGAAGAACTTTGTAATGCACGCCCGGT

>AM261760

GAGGAAACACAAAGCGGGAGGAAAGTAACTGATA-AATCGAACGCAGTTCACTAAAATAGATGGAACAA

>AM261760

TTAAGTCGAGAACTGTTCAGCTAAACTCTTGCGCTGATAAGGTAGGTAAGAGTATTATTATTCTTACCA

>AM261760

CAAGGACGAGTTTTAGCGGCTAAAGGTGTTGACG-TGCGAGAAATGTTTAGCTAAACTTCTCTCATGTG

>AJ877225

TTATGACTTCAAACCCTCAAAAGGTGCATTTACT--CAAATCCTCAATATGCTATACTGCGCTTGAGTA

>AJ877225

CAACTCACCCGGCATGAGTCGATAAGACTTGATG-TAACTACAAAAAGACGCTACAATAAGAACACAAT

>AJ877225

AAGCGTCTCTGCCAGTGGAGCGTGCCCATTGACT-GAAACTGAGAGACGGGCTTATCTAACTTTCCTGT

>AJ877225

CTGGCTCATCCTGCAAGGGGCAAGCCCCTTGACC-CCGGACAGTGCGGCGCTTCGCTTCTTACTTTCCA

>AJ877225

ATCAGCAGTTTAGGTGCACGGGACGTGCTTTGCA-ACAAGCATAAAAGGCTCTATGCTGTCAACTCCGC

>AJ877225

TGGCCGACTAATGATCGTTTGACGGACATTGACG-GTAAGGCATTTTTGCATTACTATGTGAAGCATCA

>AJ877225

CCATGCCGGACACTGTCCAGCAAAGTTATTGCAC-AACGGGTTGTCGGACATTAAGATAGGCGGACGGA

>AJ877225

AAGGCATTGCGATTCTCGAAAATGGTTCTTGAAA-TTCTATTCTTGATTGCATATCATCTCAACGAGTT

>AJ877225

CGAAAACCGCTCGCATATCGTATCTGTATTGCGT--ATCATATCTGTATTGATACGATTTACACTGATA

>AJ877225

TTTACGACACCTCCTTGAGGTTGCCGCGTTTACATCATTACCTGAACAGGTATAAAATTCCGTCATGAC

>AJ877225

TGTTTGGAACCCCAAGGCGTGACCGATGCAGACG-ACCAGAAACACCCAAACTATGGTCTGCATTGTAG

>KX711879

AAACATGTAAATAAATTCCGTACTTGTATTGACT-TTAAAGGCGTAATCACCGATAATTAAATCATCGG

>KX711879

AATTAGGTAAATATAATCCGTACTTGTATTGACT-TTAACGCCCCATTGTACGATAATTAAAAAGTGGC

>KX711879

AATAAAGTTTATAAAAATCGTACTTGTATTGACC-CCAGTTTATTCTGAGTATATCCTTTCTGTAACCA

>KC543497

GTCAGGATAGGATTGAATTTTGAATTTATTGACA-TATCTCGTTGAAGGTCATAGAGTCTTCCCTGACA

>PAU12338

GTCAGAATAGAGTTAAATTTCCTATTGATTGACA-TATTCCGTCAAAGGTAATAGATTTCATCCTGACA

>PAU12338

TCCGGCGTTGGGCGCACAATAAGGCTCCTTGCAG-AGTTGCTTGAAAGTTGTTACGATTCAAATTCAAT

>PAU49101

CATCGCACACTCAGCATGGCCTGTTGAGTTGCAT--CTAAAATTGACCCACTTAGGGTAAAGATTTGCG

>PAU49101

TGCACCAGTTGATTGGGCGTAATGGCTGTTGTGCAGCCAGCTCCTGACAGTTCAATATCAGAAGTGATC

>AJ867811

TCAGGTGTTGGGCGCACAATAAGGCTCCTCGCTG-AGTTGCTTGAAAGTTGTTACGATTCAAATTCAAT

>AJ786649

GTCAGAATAGAGTTGCCTTCCGAATTGATTGACA-TGCGCCGTCAAGGGTCATAGATTTCTTCCTGACA

>AB104852

TCAAACATCGACCCACGGCGTAACGCGCTTGCTGCTTGGATGCCCGAGGCATAGACTGTACAAAAAAAC

>AF313472

GTGGAAACGGATGAAGGCACGAACCCAGTGGACA-TAAGCCTGTTCTGTTCGTAAGCTGTAATGCAAGT

>AJ634050

CGACAAATAGAGTTGCCTTCCGAATTGATTGACA-TGCGCCGTCAAGGGTCATAGATTTCTTCCTGACA

>FN397628

GTAACGGCGCAGTGGCGGTTTTCATGGCTTGTTA-TGACTGTTTTTTTGGGGTACAGTCTATGCCTCGG

>DQ522237

CGTGGAAACGGATGAAGGCACGAACCCAGTGGACATAAGCCTGTTCGGTTCGTAAGCTGTAATGCAAG

>DQ522237

AAACATCGACCCACGGCGTAACGCGCTTGCTGCT--GTGGATGCCCGAGGCATAGACTGTACAAAAAAA

>DQ522233

GTGGAAACGGATGAAGGCACGAACCCAGTTGACA-TAAGCCTGTTCGGTTCGTAAGCTGTAATGCAAGT

>AY257539

GCCGGGTGACGCACACCGTGGAAACGGATGAAGG-CACGAACCCAGTGGACATAAGCCTGTTCGGTTCG

>GU354325

CAAACATCGACCCACGGCGTAACGCGCTTGCTGC--TTGGATGCCCGAGGCATAGACTGTACAAAAAAA

>AY294333

GTGGAAACGGATGAAGGCACGAACCCAGTTGACA-TAAGCCTGTTCGGTTCGTAAACTGTAATGCAAGG

>AF051692

CCGATGTGCGCAGCCCGGCGGATTCGGCTTGCGACGAAGCGGAAAGATCGTGAATACTGGCACGATTGC

>AF051692

TTTACATTTGGCCTGGATGTAAATGATCTTTACT--CTCTTTTGTTAATGATTATCATCCGTGCCGATC

>PAU38230

AATGGAGCAACTGGCTCTGGCTGTTGAGTTGCAT--CTAAAATTGACCCACTTAGGGTAAAGATTTGCG

>X75761

ACTGTATTCGGCTGCAACTTTGTCATGCTTGACA--CTTTATCACTGATAAACATAATATGTCCACCAA

>X75761

CGCGGATTCTTTATCACTGATAAGTTGGTGGACATATTATGTTTATCAGTGATAAAGTGTCAAGCATGA

>MH463250

GATGAAATCAATGATTTATCAAAAATGATTGAAAGGTGGTTGTAAATAATGTTACAATGTGTGAGAAGC

>PAU12891

AAAGGTGAGATTGGTTATTTCTTCGTAATTGACA-ATCATTATCATTCAACATAATTTGTTGCGCCATG

>PAU70046

CGCCCCCTCGCGGCACAGGCGCGCCACTTTGCCGCGCGCTAGCGGCCAGCCGTATAATCAGCGGTTTTC

>AY920928

GATACTTTCGGCAACCTGGTAAACGGACTTGGCC-AAATCAATTGCAATGCGTTTCATCTTCACTCTCC

>AF074954

GGATGATAAGTTTATCACCACCGACTATTTGCAA-CAGTGCCAACGCCGGGTTATTCTTATTTGTCGCT

**4 *P. putida* promoter sequences retrieved from NCBI Nucleotide**

>KY883660

GTGGAAACGGATGAAGGCACGAACCCAGTGGACA-TAAGCCTGTTCGGTTCGTAAGCTGTAATGCAAGT

>KY883660

GTGGAAACGGATGAAGGCACGAACCCAGTGGACA-TAAGCCTGTTCGGTTCGTAAACTGTAATGCAAGT

>AY907717

GTGGAAACGGATGAAGGCACGAACCCAGTGGACA-TAAGCCTGTTCGGTTGGTAAGCTGTAATGCAAGT

>AY907717

TCAAACATCGACCCACGGCGTAACGCGCTTGCTG-CTTGGATGCCCGAGGCATAGACTGTACAAAAAAA

>KP754012

GTAACGGCGCAGTGGCGGTTTTCATGGCTTGTTA-TGACTGTTTTTTTGGGGTACGTCTATGCCTCGGG

>KP754012

ATCAAACATCGACCCACGGCGTAACGCGCTTGCT-GCTTGGATGCCCGAGGCATAGACGTACCCCAAAA

>AF031895

CCCGGTCGATGCCATCGCCGCCGCCCTGTTGCTG--CAAGGCTGGCTGGAGGCCAATACCTGATCATCT

>AF031895

ATCCGTCCGAATGTGCTCGGTGCCACCCTGTCGCTGGCCGCCCATGAACGGGTAAAATTGACCGGACCC

>AY138113

ACAGGATTTGCTCATGATACGACTCCATTTGAAC-AATGTTGTGGTACCATTTAAAACTATAAAGCTAC

>AY138113

GTTCATTTCTGATCCGCGCAAACCATATTTGACC-TGACTGTCAATTGCGCCTATGGTTAATCAATAA

>AF327064

GTGGAAACGGATGAAGGCACGAACCCAGTTGACA-TAAGCCTGTTCGGTTCGTAAACTGTAATGCAAGT

>KP754011

TCAAACATCGACCCACGGCGTAACGCGCTTGCTG-CTTGGATGCCCGAGGCATAGACTGTACAAAAATA

>KP754009

TCAAACATCGACCCACGGCGTAACGCGCTTGCTG-CTTGGATGCCCGAGGCATAGACTGTACCCCAAAA

>AF321092

TGCCCATGCCGCCCTTGATGCGGAATCAATGACAGGCCATTGTCGGACTATTAATAACCACTGATGGTC

>AB062597

GTCAGAATAGAGTCGCCTTTTGCATTCTTTGACA-CCTGCTTGCCAAGGTCATAGATTTTAGCCTGACA

>PSE4653RE

AGTCAGAATAGAGTCATCTTTCGCATTTTTGACA-CATGCCTGCGAAGGTCATAGATTTCAGCCTGACA

>PSECAMRD

ACATTTGCGCCGTTTTTAAACGAAGATGTTGACC-ACACTCCTTCTCGCCAATATGCTCAGTATATCGC

>AB025418

TGCCAGCATTGACATTTTCTTCACTGTGGAACTT--GTACCGTCGTTCCAGGTCAATGTTGGCAGCGCC

>AM283489

GTGGAAACGGATGAAGGCACGAACCCAGTTGACA-TAAGCCTGTTCGGTTGGTAAACTGTAATGCAAGT

>AB077820

GATTTCGTAGTAGATTACATAGTATGTATTTACATACTAAATTTGATTGATGTAAAGCCAGTTAAAAGC

>D88554

CGGTACGGTCGATGGCGCTAGGCATGTCTTGCCCTCCATAGCCTGTTCTTGTTGTTTTTATGTCAGTGA

**5 *P. aeruginosa* background sequences**

For the training of *SAPPHIRE*, 16,000 background sequences were randomly extracted from the *Pseudomonas aeruginosa* strain PA01, accession number NC_002516

**REFERENCES**

Domínguez-Cuevas P., Marqués S. (2004) Compiling Sigma-70-Dependent Promoters. In: Pseudomonas. Springer, Boston, MA, p. 319-343
